# Dynamic functional reorganization in post-stroke aphasia: a state-of-the-art fMRI review from disease evolution to intervention

**DOI:** 10.3389/fnins.2026.1866063

**Published:** 2026-06-17

**Authors:** Luyao Xu, Lian Tang, Anji Zheng, Yuexiu Li, Yumei Zhang

**Affiliations:** 1 Department of Neurology, Beijing Tiantan Hospital, Capital Medical University, Beijing, China; 2 Department of Rehabilitation Medicine, Beijing Tiantan Hospital, Capital Medical University, Beijing, China; 3 Beijing Key Laboratory of Translational Medicine for Cerebrovascular Disease, Center of Stroke, Beijing Institute for Brain Disorders, Beijing, China; 4 China National Clinical Research Center for Neurological Diseases, Beijing, China

**Keywords:** brain network, fMRI, functional reorganization, language network, neuroimaging biomarkers, post-stroke aphasia, remodeling patterns

## Abstract

Post-stroke aphasia (PSA) is a common and disabling consequence of stroke, characterized by substantial heterogeneity in language impairment and recovery trajectories. In recent years, functional magnetic resonance imaging (fMRI) has markedly advanced our understanding of the neural mechanisms underlying PSA recovery by revealing dynamic changes in regional activation, functional connectivity, and large-scale network coordination. This review provides a state-of-the-art synthesis of fMRI studies on functional reorganization in PSA across two intersecting dimensions: the temporal evolution of the disease course (acute, subacute, and chronic stages) and the major categories of rehabilitation intervention, including behavioral therapies, neuromodulation, and combined treatment approaches. Current evidence suggests that PSA recovery is a stage-dependent and network-based neuroplastic process. In the early phase after stroke, recovery is strongly influenced by the rapid recruitment of domain-general cognitive control systems, whereas later recovery is increasingly shaped by the reorganization of residual left-hemisphere language networks, with contralesional and cerebellar contributions becoming more prominent in patients with extensive left-hemisphere damage. However, important controversies remain regarding the functional role of right-hemisphere activation and the relative significance of perilesional restoration versus compensatory recruitment. Furthermore, the field is limited by substantial heterogeneity in patient characteristics, lesion profiles, study design, and imaging methodology, which constrains comparability across studies and hinders clinical translation. By integrating current evidence, unresolved debates, and emerging trends, this review highlights key research gaps and outlines future directions for biomarker-guided, stage-adapted, and individualized rehabilitation strategies in PSA.

## 1 Introduction

Stroke represents the second leading cause of mortality and the third leading cause of physical impairment globally. Approximately 80 million individuals worldwide live with stroke-related impairments, including roughly 2,445,000 new cases annually (Gu et al., 2021). Aphasia is one of the most debilitating consequences of stroke, affecting more than one third of stroke survivors (Breitenstein et al., 2017; Palmer et al., 2019), particularly those with middle cerebral artery lesions in the language-dominant hemisphere (Flowers et al., 2013). This disorder affects not only the comprehension and production of language, but also reading and writing skills, leading to a significant reduction in quality of life and social engagement (Stockert et al., 2020). Despite many aphasic exhibiting spontaneous partial language recovery, difficulties persist into the chronic phase in at least 40% of initially aphasic patients (Stefaniak et al., 2022). There is, therefore, an urgent need to understand the mechanisms involved in language recovery so that we can develop targeted treatments for this costly and debilitating condition.

Functional magnetic resonance imaging (fMRI) has become one of the most important non-invasive tools for studying the neural basis of PSA. By characterizing changes in blood oxygen level-dependent (BOLD) signals during language tasks or at rest, fMRI enables researchers to visualize evolving activation patterns, functional connectivity, and cross-network coordination associated with language impairment and recovery (Yang et al., 2020; Du et al., 2024). Compared with conventional structural imaging, fMRI offers a dynamic systems-level perspective on post-stroke neuroplasticity and provides a valuable bridge between basic neuroscience and clinical rehabilitation.

Although a growing body of fMRI studies has partly elucidated the neural mechanisms of language recovery in PSA, the current literature remains constrained by several important limitations. Many studies are based on relatively small samples, single-center designs, and heterogeneous patient populations. In addition, variability in aphasia subtype, lesion location, post-stroke stage, task paradigms, imaging analysis pipelines, and intervention protocols has reduced comparability across studies and complicated interpretation. Moreover, the neural effects of different rehabilitation approaches—such as intensive language therapy, non-invasive brain stimulation, and combined interventions—have rarely been systematically integrated within a unified framework.

At the same time, the field has advanced substantially in recent years. Beyond early lesion-deficit accounts, contemporary fMRI research increasingly conceptualizes PSA as a disorder of distributed network disruption and dynamic reorganization. Studies now examine not only focal regional activation but also resting-state functional connectivity, dynamic network interactions, domain-general control systems, cerebellar contributions, and treatment-related remodeling.

Therefore, this review aims to provide a state-of-the-art synthesis of fMRI-based evidence on functional reorganization in PSA across two intersecting dimensions: the temporal progression of recovery (acute, subacute, and chronic stages) and the effects of rehabilitation intervention (behavioral therapies, neuromodulation, and combined or adjunctive treatments). By integrating regional activation patterns, network-level functional connectivity, theoretical models, competing interpretations, and clinical implications, this review seeks to provide a systematic neuroimaging perspective on the multi-stage neuroplastic mechanisms underlying PSA recovery and to inform more precise, stage-adapted, and individualized rehabilitation strategies. Furthermore, because methodological variability strongly influences fMRI findings in stroke populations, this review also evaluates key methodological issues across the aphasia imaging literature, including fMRI modality, task paradigms, acquisition and preprocessing approaches, connectivity metrics, parcellation reliability, lesion handling, and motion artifact control.

## 2 Search strategy and selection criteria

This article is a narrative review of the literature on dynamic functional reorganization in post-stroke aphasia using fMRI. To identify relevant studies, we searched PubMed, Embase, Cochrane library and Web of science databases for articles published between January 1990 and January 2026. The search terms included combinations of the following keywords: “aphasia,” “post-stroke aphasia,” “functional magnetic resonance imaging,” “fMRI,” “network,” “brain network,” “functional connectivity,” “functional activity.” After removing irrelevant and duplicate records, we performed a preliminary screening by reading the titles and abstracts, and excluded review articles, meta-analyses, case reports, and conference abstracts. The full texts of the remaining articles were then reviewed. We included original research studies that clearly described the study population, research methods, and sample size. Exclusion criteria were: case reports, animal studies, conference abstracts, and studies not using fMRI as the primary imaging modality. Given the narrative nature of this review, we did not perform a formal quality assessment or meta-analysis. The selection of cited articles was based on their relevance to the key themes of this review, with the goal of providing a balanced synthesis of current evidence, major controversies, and research gaps.

## 3 Methodological considerations in fMRI studies of post-stroke aphasia

Although fMRI is central to investigating functional reorganization in PSA, methodological heterogeneity across studies complicates interpretation. Variability in fMRI modality, task design, acquisition parameters, preprocessing pipelines, connectivity metrics, parcellation strategies, and motion control can substantially influence reported activation and network findings, particularly in stroke populations with focal lesions, altered neurovascular coupling, and increased motion susceptibility.

### 3.1 fMRI modalities and acquisition parameters

Most PSA studies use task-based fMRI, resting-state fMRI, or both. Task-based fMRI identifies residual or compensatory language-related activation; resting-state fMRI characterizes functional connectivity within and between brain networks. Data are typically acquired with EPI sequences on 3-T scanners. Common parameters: TR 1.5–3.0 s, TE 25–40 ms (3-T), flip angle 70–90°, isotropic voxels 2–4 mm. Resting scans last 5–10 min; task runs vary. High-resolution T1 images are obtained for anatomy and lesion delineation (Koc et al., 2026; Xu et al., 2023). Unfortunately, acquisition parameters are often under-reported, limiting reproducibility. Studies should report scanner strength, sequence type, TR/TE, voxel size, volumes, scan duration, slice coverage, multiband use, and distortion correction.

### 3.2 Task paradigms

Common tasks include picture naming, verb generation, semantic/phonological judgment, auditory comprehension, sentence completion, reading, repetition, and lexical decision (Liuzzi et al., 2024; Thomas et al., 2023; Sánchez et al., 2025). These differ in cognitive demands, engaging overlapping but non-identical networks. Block designs offer stronger power but cannot separate correct/incorrect trials; event-related designs allow trial-level modeling but require many trials (Lei et al., 2024). Overt speech tasks are ecologically valid but produce motion artifacts; covert tasks reduce motion but complicate behavioral monitoring (Zhang W. et al., 2024). Interpretation must account for task performance: reduced activation may reflect dysfunction or poor engagement; increased right-hemisphere activation may be compensatory or inefficient (Knights et al., 2021).

### 3.3 Preprocessing pipelines and software

Preprocessing is essential for reducing non-neural noise and artifacts in fMRI, especially in post-stroke aphasia where lesions, motion, and vascular alterations may bias results (Lamouroux et al., 2025). Common software includes SPM, FSL, AFNI, CONN, DPABI/REST, FreeSurfer, ANTs, and fMRIPrep (Di and Biswal, 2022; McAvoy et al., 2025; Waller et al., 2022). Initial volumes are discarded to reach magnetization steady state. Slice-timing correction reduces inter-slice temporal offsets, and motion correction realigns images to minimize head-movement artifacts, which is critical for patients with hemiparesis or overt speech. Co-registration aligns functional with structural images for accurate localization. Spatial normalization enables group-level inference, cost-function masking, or enantiomorphic normalization are recommended to prevent bias. Smoothing improves signal-to-noise ratio but may blur lesion boundaries (Kristanto et al., 2024; Xu et al., 2023, 2026a). For resting-state fMRI, nuisance regression of motion, white matter, and cerebrospinal fluid signals, combined with band-pass filtering, reduces physiological noise (Xu et al., 2023). Global signal regression remains controversial as it may introduce artificial negative correlations. For task-based fMRI, the general linear model is standard, but altered neurovascular coupling after stroke may affect the hemodynamic response (Braban et al., 2023). Each preprocessing step is crucial to ensure that subsequent analyses reflect true neural reorganization rather than residual artifacts.

### 3.4 Connectivity metrics and network analysis

Seed-based correlation is widely used, with seeds in left inferior frontal, temporal, and parietal regions and their right-hemisphere homologues. ROI correlation matrices, independent component analysis, and graph-theoretic measures (e.g., clustering coefficient, global efficiency) are also applied (Yang et al., 2020). Effective connectivity methods like dynamic causal modeling (DCM) and Granger causality (GC) estimate directional neural interactions. DCM quantifies how one region causally influences another and how experimental context modulates this influence (Jiang et al., 2025). GC tests whether past activity predicts future activity (Han et al., 2023). In PSA, DCM reveals that successful language recovery depends on restored top down control from left inferior frontal to left posterior temporal regions and on context dependent interhemispheric shifts. GC remains unexplored in PSA, representing a key future direction. Both methods require careful model specification, adequate signal to noise ratio, and handling of lesion induced hemodynamic changes. Integrating effective connectivity with functional and structural (DTI) measures enables a causal understanding of network reorganization in PSA.

### 3.5 Brain parcellation and ROI reliability

Atlas choice affects results. Commonly used atlases: AAL, Harvard-Oxford, Brainnetome, Schaefer/Yeo, Glasser (Khan and Shang, 2025; Makris et al., 2023; Yan et al., 2023; Qu et al., 2025; Lu et al., 2024). Anatomical atlases are easy to compare but may not align with individual functional regions; functional or connectivity-based parcellations better capture network organization but may be unreliable in patients with large lesions. Studies should report the parcellation used, handling of lesioned voxels, ROI coverage, and whether analyses were in native or normalized space.

### 3.6 Motion artifacts and quality control

Motion is a major concern due to hemiparesis, fatigue, or overt speech tasks. Even small movements cause spurious activation and distort connectivity. Strategies include foam padding, practice, shorter runs, and censoring high-motion volumes (framewise displacement thresholds typically 0.2–0.5 mm). Motion parameters should be included as nuisance regressors, and group motion differences tested. For resting-state data, scrubbing or ICA-based denoising is helpful (Parkes et al., 2018).

### 3.7 Implications for interpretation

Methodological variability may explain inconsistent findings about left-hemisphere restoration, right-hemisphere compensation, and domain-general recruitment. The same right inferior frontal activation may be compensatory in one study but maladaptive in another, depending on task difficulty, lesion characteristics, and connectivity with residual left language regions. Future studies should adopt transparent reporting: patient characteristics, aphasia severity, lesion volume, time since stroke, fMRI modality, acquisition parameters, task performance, preprocessing details, motion thresholds, parcellation, and lesion handling. Multicenter studies with harmonized protocols, longitudinal designs, and open data sharing are needed to improve reproducibility and clinical translation.

## 4 Neural mechanisms of post-stroke aphasia recovery

### 4.1 Neuroplasticity

The recovery mechanisms of PSA are closely linked to neuroplasticity. Following stroke-induced damage to language-related regions (e.g., Broca’s or Wernicke’s area), surviving neurons undergo functional reorganization via synaptic plasticity (Kiran and Thompson, 2019). This is reflected in the dynamic regulation of synaptic strength—long-term potentiation strengthens critical synaptic connections, whereas long-term depression weakens unnecessary ones, thereby optimizing neural circuitry. Concurrently, neurons in perilesional areas form new synaptic connections through axonal sprouting and dendritic structural remodeling, partially compensating for lost neural functions (Carmichael, 2006).

### 4.2 Reorganization of the left-hemisphere language network

A major route of PSA recovery involves the restoration or reorganization of residual left-hemisphere language systems, especially frontotemporal and temporoparietal regions surrounding the lesion (Nenert et al., 2018; van Oers et al., 2010). In fMRI, two main modalities are used to assess language recovery: task-based fMRI, which measures the magnitude of blood oxygen level-dependent (BOLD) signal change (e.g., percent signal change or contrast estimates) during language tasks relative to baseline, typically analyzed using general linear models to produce statistical parameter maps (*t*-values or z-scores); and resting-state fMRI, which quantifies temporal correlations of spontaneous BOLD fluctuations (functional connectivity) between brain regions, often expressed as correlation coefficients or Fisher’s z-transformed values. Drawing on these approaches, fMRI studies have repeatedly shown that improved language performance may be associated with increased activation in left perilesional cortex (reflected as higher BOLD contrast estimates or *t*-values in task-based analyses), strengthened functional connectivity among surviving language regions (measured as increased correlation coefficients or z-scores in resting-state analyses), and more efficient integration within the residual language network (Xu et al., 2020; Zhu et al., 2014; Ren et al., 2022; Xie et al., 2023). These findings support the view that the preserved left hemisphere remains the most biologically relevant substrate for successful recovery in many patients.

### 4.3 Interhemispheric competition and right-hemisphere recruitment

One influential framework for interpreting PSA recovery is the interhemispheric competition model (Di Pino et al., 2014; Gholami et al., 2022). It proposes that while both hemispheres are capable of processing language tasks, under normal conditions, the left hemisphere inhibits the activity of the right hemisphere via the corpus callosum. After a stroke, this interhemispheric inhibitory balance is disrupted, leading to hyperactivation of right-hemisphere language regions, which in turn suppress activity in the lesioned left hemisphere and its surrounding cortical areas. This model is supported by substantial neuroimaging evidence (Belopasova et al., 2020; Sheppard and Sebastian, 2021). Furthermore, clinical trials utilizing transcranial magnetic stimulation have demonstrated that inhibiting these overactive right-hemisphere homologous regions can enhance language performance in aphasic patients, with effects that are sustainable over time (Lefaucheur et al., 2020; Cheng et al., 2024; Low et al., 2023).

However, this model does not fully account for all empirical findings. A growing number of fMRI studies indicate that right-hemisphere recruitment may be beneficial, particularly in patients with severe left-hemisphere injury, limited perilesional reserve, or high processing demands (Crinion and Price, 2005; Mohr et al., 2014; Barbieri et al., 2019). Under such conditions, contralesional activity may represent adaptive compensation rather than maladaptive interference.

### 4.4 Hierarchical recovery model and lesion-dependent recovery

A more nuanced framework is provided by hierarchical recovery model (Meier et al., 2019). It suggests that language recovery in PSA patients can be categorized into three distinct patterns based on the severity of left-hemisphere damage:

➀ When the core regions of the left-hemisphere language network remain intact, the original language network can be largely restored, and the corresponding language functions can recover almost completely;
➁ When the core regions of the language network are damaged, reduced transcallosal inhibition leads to activation of perilesional cortex, followed by recruitment of secondary hubs within the left-hemisphere language network for compensatory support. However, in this scenario, language function does not fully recover;
➂ When the left-hemisphere language network is severely damaged, decreased transcallosal inhibition results in hyperactivation of contralateral homologous regions, leading to a shift of language functions to the right hemisphere. Under these conditions, language recovery is the most limited.

### 4.5 Domain-general cognitive control networks

**FIGURE 1:**
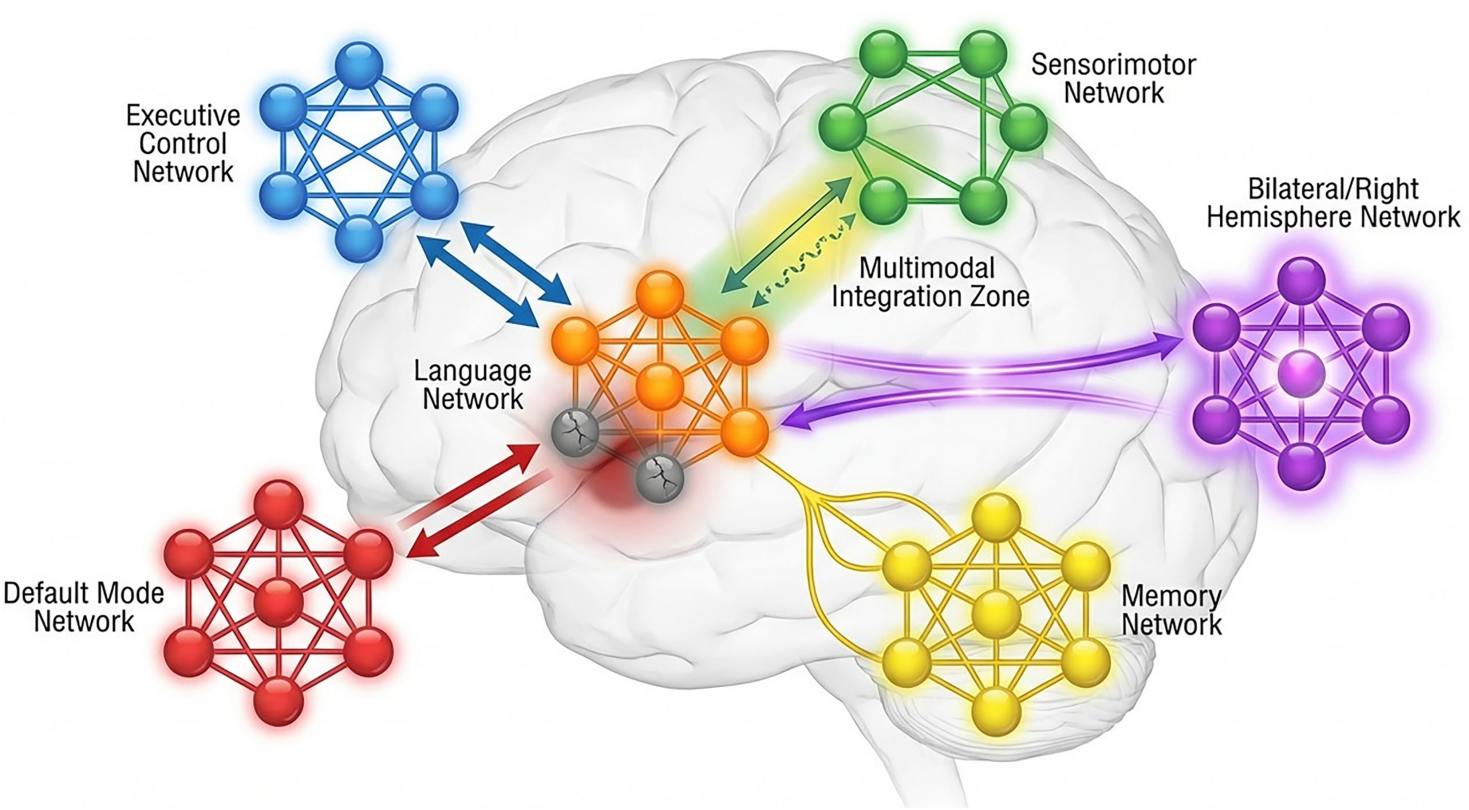
Schematic diagram of brain functional networks, showing the main language-related brain networks (sensorimotor network, executive control network, bilateral/right hemisphere network, multimodal integration zone, language network, default mode memory network).

Recovery from PSA is not mediated by language networks alone. Domain-general systems appear to play an important role in supporting impaired language function. These networks may contribute to attentional allocation, executive control, performance monitoring, working memory, conflict resolution, and learning during therapy. fMRI studies (Xu et al., 2026a,b; Fan et al., 2022; Wang et al., 2025a; Sarmukadam et al., 2025; Yang et al., 2018; Sarmukadam and Behroozmand, 2023) have shown that: when left-hemisphere language areas are damaged, the brain partially compensates by releasing interhemispheric inhibition and engaging neuroplastic adjustments, leading to enhanced activation of homologous right-hemisphere regions. Concurrently, the synergy between the default mode network (a set of brain regions active during rest and internally focused thought) and language-processing regions is disrupted, while the executive control network attempts to compensate for the language network damage by strengthening cross-hemispheric and cross-network connections. The memory network also undergoes dynamic reorganization, involving both adjustments within the language network itself and compensatory activation of non-language regions, all aimed at maintaining cognitive and communicative functions through multimodal network synergy. At the sensorimotor network level, disruptions in functional connectivity caused by left-hemisphere brain damage can induce widespread dysfunction at the network level, ultimately undermining the sensorimotor integration processes on which speech auditory feedback control depends. Furthermore, multimodal integration areas (e.g., the angular gyrus and supramarginal gyrus) exhibit changes such as functional connectivity reorganization, altered activation patterns, and reduced integrative capacity. Collectively, these holistic remodeling mechanisms reflect an adaptive process in which the brain, under aphasic conditions, recruits non-language pathways and reorganizes neural networks in an effort to restore function, thereby illustrating the whole-brain nature of language processing (see Figure 1 for other brain networks that are functionally closely connected to the language network following aphasia).

### 4.6 Subcortical and cerebellar contributions

Although many fMRI studies have focused on cortical language regions, subcortical structures and the cerebellum also contribute to PSA recovery. The cortico-basal ganglia-thalamo-cortical loop influences language by regulating automaticity, fluency, and prosody, particularly during the sequential execution of complex utterances (Thompson-Lake et al., 2022; Zhang J. et al., 2022).

Meanwhile, the cerebellum has increasingly been implicated in language production, executive control, verbal working memory, and adaptive learning. It is important to note, however, that most of this evidence derives from studies of healthy speakers or non-aphasic populations. In healthy individuals, specific cerebellar subregions have been linked to distinct functions: cerebellar lobule VI and right lobule VIIb with language function (Zhang X. et al., 2024); bilateral lobules VII and right lobule VIIIa with working memory (Stoodley et al., 2010); and right lobules VI/VII with reading (Lee et al., 2024). The cerebellum forms extensive reciprocal cerebro-cerebellar circuits with the cerebral cortex (Keser et al., 2023; Zhang X. et al., 2022).

By contrast, direct evidence for the cerebellum’s role in post-stroke aphasia recovery remains considerably more limited and preliminary. A small number of studies have reported altered cerebellar activity or connectivity in PSA patients. For instance, during language tasks in aphasic patients, activation in the right posterior cerebellum facilitates speech generation, whereas left posterior cerebellar activation may support executive control during language production (Tilton-Bolowsky et al., 2024; Sebastian et al., 2020; Xie et al., 2022). Resting-state connectivity between the right cerebellar Crus I/II and residual bilateral cerebral hemisphere regions has been shown to predict naming performance in chronic PSA (Stilling et al., 2025). Increased activation of the right cerebellar Crus I after Broca’s area damage has also been observed, suggesting a possible adaptive role within reorganized speech-production networks (Lorca-Puls et al., 2021). Nevertheless, given that most mechanistic insights into cerebellar language functions are derived from healthy populations, the specific contribution of the cerebellum to language recovery after stroke remains incompletely understood and warrants further investigation using aphasia-specific samples and multimodal imaging approaches.

### 4.7 Directed effective connectivity as a window into network reorganization

Recent DCM studies have revealed directional principles of language network reorganization after stroke. Jiang et al. (2025) conducted a longitudinal DCM study in 34 stroke patients and identified four recovery patterns: (i) early facilitatory influences from bilateral multiple-demand regions to left-hemisphere language areas, predicting later language gains; (ii) within-network shifts from bottom-up (subacute) to restored top-down modulation (chronic); (iii) acute-phase facilitatory right-to-left inferior frontal connectivity in frontal-lesion patients; and (iv) lesion-dependent substitution of multiple-demand nodes (right dorsolateral prefrontal cortex for frontal lesions, supplementary motor area for temporo-parietal lesions). These directional changes correlated with behavioral improvement.

Sarmukadam et al. (2025) used DCM during a speech task in chronic aphasia and found increased self-inhibition in left precentral gyrus (impaired feedforward commands), reduced precentral-to-inferior parietal connectivity, and compensatory right precentral-to-right superior temporal connectivity.

Granger causality has been rarely used. Han et al. (2023) applied it to resting-state fMRI in acute aphasia, showing that increased connectivity from right frontal to left occipital and from right temporal to cerebellum positively correlated with language scores. Task-based Granger studies are lacking. These findings demonstrate that aphasia recovery involves dynamic, directional network reorganization, highlighting DCM and GC as tools for identifying stage- and lesion-specific neuromodulation targets.

## 5 Dynamic evolution of functional reorganization across disease stages

### 5.1 Acute phase (within 2 weeks)

The acute phase of post-stroke aphasia is characterized by an unstable brain state in which widespread functional suppression coexists with the earliest compensatory responses. Neuroimaging studies consistently show markedly reduced activation in classical left-hemispheric language regions during language tasks, with only weak engagement of areas such as the left inferior frontal gyrus compared with healthy individuals (Saur et al., 2006; Nenert et al., 2018). At the same time, early compensatory activity is already detectable in a limited set of regions, including the right inferior frontal gyrus, contralateral hippocampal/parahippocampal areas, and the left posterior cerebellum (Wang et al., 2025a; Yang et al., 2016). Increased activation of the pre-supplementary motor area/dorsal anterior cingulate cortex, a key node of the cingulo-opercular control network (a domain-general network involved in task set maintenance and sustained attention), has also been observed during this stage and independently predicts subsequent language recovery, suggesting early involvement of domain-general cognitive control systems in language restoration (Geranmayeh et al., 2017).

Alongside this generalized suppression, the acute phase shows an emerging shift toward bilateral recruitment. Enhanced activation and functional connectivity in right-hemispheric language-related regions have been reported, and stronger intra-right-hemispheric connectivity is associated with less severe aphasia (Yang et al., 2016; Guo et al., 2019; Meier et al., 2023). Right-hemispheric domain-general systems may further support early comprehension recovery by facilitating cognitive resource allocation and sequential information processing (Schneider et al., 2022). This pattern of bilateralization appears to depend partly on lesion location, with frontal lesions more likely to induce early recruitment of right-hemispheric homologues, whereas temporoparietal lesions engage broader bilateral domain-general networks (Stockert et al., 2020; Jiang et al., 2025).

**FIGURE 2:**
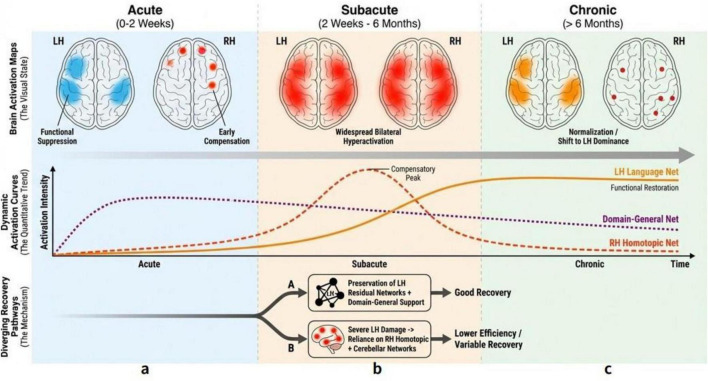
Dynamic evolution of functional reorganization across disease stages. Acute phase **(a)**: Marked by widespread functional network disruption, featuring suppressed activation in core left-hemisphere language areas (blue). Concurrent early compensation involves heightened activation in contralateral right-hemisphere regions (red). Overall global functional connectivity is broadly weakened. Subacute phase **(b)**: Characterized by bilateral widespread “hyperactivation.” Right-hemisphere homologous areas show peak activation (red), and compensation involves both these regions and bilateral domain-general networks. Functional reorganization of residual left-hemisphere networks also begins. Chronic phase **(c)**: Characterized by stabilization and rebalancing, with activation patterns partially normalizing toward left-hemisphere dominance (orange). The right hemisphere providing conditional, functionally specific support.

At the whole-brain level, however, the dominant feature of the acute stage remains widespread network suppression. Functional connectivity is reduced both within the language network and between language regions and other large-scale systems, including sensorimotor and attention networks (Wang et al., 2014, 2025a; Xu et al., 2020; Song et al., 2024; Han et al., 2023; Nair et al., 2015). Graph-theoretical analyses further demonstrate reduced global and local efficiency, indicating disrupted information transfer across the brain (Chen Q. et al., 2021). This impairment reflects not only local tissue damage but also remote functional disconnection, whereby structurally intact regions connected to the lesion become functionally inhibited (Wawrzyniak et al., 2022). In parallel, the cerebellum appears to be recruited early in recovery: increased spontaneous activity in the left posterior cerebellum, greater dynamic variability within cerebellar networks, and strengthened connectivity between the right Wernicke’s homologue and the cerebellar vermis have all been linked to recovery potential (Wang et al., 2025a; Han et al., 2023; Xu et al., 2023). Thus, the acute phase should be viewed not only as a period of maximal impairment, but as a diagnostic window in which the presence of domain-general and cerebellar engagement can help identify patients likely to recover spontaneously. Overall, the acute stage is marked by extensive disruption of the language network, accompanied by the initial recruitment of right-hemispheric, cerebellar, and domain-general fMRI control systems that may lay the foundation for subsequent reorganization (The functional reorganization in the acute phase is shown in Figure 2a and Table 1).

**TABLE 1:** Stage-specific patterns of functional reorganization during recovery from post-stroke aphasia.

Recovery stage	Time window	Main fMRI features	Dominant neural mechanisms	Typical hemispheric pattern	Clinical significance
Acute stage	Within 2 weeks	Reduced activation in language-related regions; decreased network stability; disrupted functional connectivity; possible diaschisis-related suppression	Early network inhibition; functional disconnection; initial compensatory recruitment of spared regions	Predominantly reduced left-hemisphere activity with variable early contralesional recruitment	May reflect the earliest neural substrate for spontaneous recovery
Subacute stage	From 2 weeks to 6 months	Widespread bilateral activation; dynamic interhemispheric rebalancing; increased recruitment of right-hemisphere homologues; reorganization of language and control networks	Peak neuroplasticity; compensatory recruitment; network reconfiguration; enhanced cross-network interaction	Bilateral activation is common, often with prominent right-hemisphere involvement	Considered a critical window for rehabilitation and treatment-induced plasticity
Chronic stage	After 6 months	More stable activation patterns; re-engagement of residual left perilesional regions; selective compensatory support from contralesional and domain-general networks	Stabilization of reorganized networks; partial normalization; individualized compensation	Relative shift toward more efficient left-hemisphere processing in successful recovery, with context-dependent right-hemisphere support	May provide biomarkers for prognosis, treatment targeting, and long-term rehabilitation planning

fMRI, functional magnetic resonance imaging.

### 5.2 Subacute phase (from 2 week to 6 months)

During the subacute phase of post-stroke aphasia, brain function undergoes dynamic reorganization characterized by widespread bilateral hyperactivation, compensatory right-hemisphere recruitment, and progressive restoration of left-hemispheric language networks. A common finding across neuroimaging studies is extensive bilateral activation exceeding normal levels, particularly in the right inferior frontal gyrus and supplementary motor area, with this early hyperactivation often associated with initial language improvement (Saur et al., 2006; Stockert et al., 2020; Fan et al., 2022; Nenert et al., 2018; Qiu et al., 2017; Li et al., 2023; Gu et al., 2025). This pattern likely reflects an early compensatory response in which the brain mobilizes broadly distributed neural resources to support residual language function after injury. Importantly, this phase represents the peak of neuroplasticity, where the direction of reorganization—whether toward left-hemisphere restoration or persistent reliance on bilateral circuits—can be favorably modulated by timely intervention.

At the same time, the right hemisphere appears to play an important compensatory role during this stage. Increased activation and altered connectivity have been observed in right-hemispheric homologues and domain-general regions, including the inferior frontal gyrus, prefrontal cortex, supramarginal gyrus, supplementary motor area, and cerebellum, and these changes are frequently associated with better language performance (van Oers et al., 2010; Balaev et al., 2016; Wang et al., 2014; Yang et al., 2016; Xu X. et al., 2021; Xie et al., 2022; Meier et al., 2023). In particular, persistent upregulation of the right supplementary motor area following damage to the left extreme capsule has been linked to recovery of language comprehension, highlighting the contribution of right-hemispheric domain-general systems to early compensation (Schneider et al., 2022).

Despite this contralesional support, recovery in the subacute phase also depends critically on the preservation and reorganization of residual left-hemispheric language networks. Studies consistently show that structural integrity, increased activation, and strengthened intrahemispheric connectivity within left frontal and temporal language regions are associated with better recovery of naming, comprehension, and other language functions (van Oers et al., 2010; Nenert et al., 2018; Xu et al., 2020; Zhu et al., 2014; Ren et al., 2022; Xie et al., 2023; Wang et al., 2014). Together, these findings suggest that subacute recovery reflects a transitional reorganization process in which early bilateral and right-hemispheric compensation coexists with, and may gradually give way to, restoration of more efficient left-hemispheric language network function (The functional reorganization in the subacute phase is shown in Figure 2b and Table 1).

### 5.3 Chronic phase (after 6 months)

During the chronic phase of post-stroke aphasia, language recovery typically reaches a relative plateau, and brain network reorganization becomes more stabilized, forming a relatively consistent compensatory architecture. Overall, chronic-stage recovery is characterized by partial normalization of activation patterns, increasing reliance on preserved left-hemispheric language networks, context-dependent recruitment of right-hemispheric resources, cerebellar–cerebral circuit remodeling, and rebalancing of large-scale network segregation and integration.

A consistent finding across observational studies is that language activation gradually shifts from the widespread bilateral pattern often observed in the acute and subacute phases toward a more left-lateralized organization in the chronic stage (Saur et al., 2006; Stockert et al., 2020; Fan et al., 2022; Wawrzyniak et al., 2022; Jiang et al., 2025). This pattern is consistent with the “three-phase recovery model,” in which activation peaks return to left-hemispheric language regions during later recovery (Saur et al., 2006). Whole-brain dynamic analyses further suggest that chronic-stage brain activity becomes more flexible and increasingly resembles that of healthy controls (Fan et al., 2022). Effective recovery, however, depends strongly on the structural preservation and functional reorganization of residual left-hemispheric networks. Integrity and activation of key regions such as the inferior frontal gyrus, middle temporal gyrus, angular gyrus, supramarginal gyrus, and superior temporal sulcus are reliable predictors of language outcomes (Sims et al., 2016; Griffis et al., 2017a,b; Skipper-Kallal et al., 2017). Patients with better recovery tend to show a more left-lateralized and normalized activation pattern, whereas excessive dependence on right-hemispheric activation is often associated with poorer outcomes (Szaflarski et al., 2013).

The role of the right hemisphere in chronic aphasia remains complex and highly context dependent. In patients with extensive left-hemispheric damage or during tasks requiring complex semantic integration and syntactic processing, activation of right-hemispheric homologues, including the right inferior frontal gyrus, superior temporal gyrus, and anterior superior temporal sulcus, may support compensatory language processing and correlate with behavioral improvement (Crinion and Price, 2005; Mohr et al., 2014; Barbieri et al., 2019). However, right-hemispheric recruitment can also reflect inefficient or maladaptive processing. For example, right inferior frontal activation has been associated with erroneous naming responses, and enhanced activation of right dorsal inferior frontal regions has shown limited predictive value for language recovery (Postman-Caucheteux et al., 2010). Thus, right-hemispheric activity is not uniformly beneficial; its contribution depends on lesion distribution, task demands, and the availability of residual left-hemispheric resources.

Cerebellar–cerebral circuit plasticity represents another important feature of chronic-stage reorganization. Damage to left frontal language regions can disrupt the typical left frontal–right cerebellar loop and lead to recruitment of a compensatory right frontal–left cerebellar pathway (Connor et al., 2006). Resting-state studies further show strengthened connectivity between the right cerebellar Crus I/II and left-hemispheric language regions, such as the left superior temporal gyrus, and this enhanced connectivity independently predicts naming performance (Stilling et al., 2025). Increased activation of the right cerebellar Crus I after Broca’s area damage also suggests that the cerebellum may serve as an adaptive compensatory node within reorganized speech-production networks (Lorca-Puls et al., 2021).

At the whole-brain level, successful chronic recovery involves a new balance between functional segregation and integration. Increased resting-state modularity or longer dwell time in states with reduced between-network coupling has been associated with improved narrative performance, suggesting that moderate network segregation supports efficient language processing (Duncan and Small, 2016, 2018). At the same time, language function also requires integration between the language network and domain-general systems. Dynamic connectivity studies show that temporal properties of modular network states correlate with language function, while connectivity between the language network and executive control network remains behaviorally relevant (Xu et al., 2026a). Moreover, recovery involves multi-stage reorganization of interactions between language networks and multiple-demand systems, including attention and executive control networks; strong early support from these systems may help maintain performance, whereas later attenuation or refinement of control-like connectivity is associated with better long-term outcomes (Houldin et al., 2025; Jiang et al., 2025).

In summary, chronic-stage post-stroke aphasia recovery reflects a multi-scale, network-based adaptive process rather than a simple shift between left-hemisphere restoration and right-hemisphere compensation. Successful recovery is grounded in the preservation and reorganization of residual left-hemispheric networks, supported by selective right-hemispheric recruitment, cerebellar–cerebral circuit remodeling, and global optimization of network segregation and integration (The functional reorganization in the chronic phase is shown in Figure 2c and Table 1).

## 6 fMRI findings across intervention modalities

### 6.1 Behavioral language interventions

➀ Intensive language therapy

Functional magnetic resonance imaging studies indicate that intensive language therapy promotes post-stroke aphasia recovery through dynamic, multi-level neural reorganization involving local restoration, network-level plasticity, and compensatory recruitment. At the regional level, treatment benefits are associated with reactivation of dysfunctional left perilesional language areas, supporting the reintegration of residual language functions within the ipsilesional hemisphere (Meinzer et al., 2008). This reorganization appears to evolve over time: short-term gains after intensive naming training are linked to increased activation in memory- and attention-related regions, including the bilateral hippocampus and precuneus, whereas long-term maintenance relies more on left perilesional regions and right temporal homologues (Menke et al., 2009).

At the network level, both pre-existing brain organization and treatment-induced plasticity recovery. Higher baseline resting-state global efficiency predicts better language outcomes, suggesting that stronger network integration provides a favorable substrate for rehabilitation. Following therapy, improvements in language and attention are accompanied by enhanced connectivity among language-related and domain-general networks, including default mode, auditory, salience (a system that detects behaviorally relevant stimuli and switches between large-scale networks), and visual systems (Menke et al., 2009). These findings suggest that successful recovery depends not only on reorganization of core language circuits but also on improved coordination with broader cognitive networks.

Right-hemisphere recruitment also contributes to recovery, particularly in chronic aphasia or when left-hemisphere damage is extensive. Intensive language therapy, including constraint-induced language therapy, treatment of underlying linguistic forms, melodic intonation therapy, and sentence-level interventions, have been shown to increase activation in right inferior frontal, superior temporal, posterior inferior frontal, superior parietal, and angular regions, with these changes supporting sentence processing, syntactic comprehension, speech fluency, and story comprehension (Mohr et al., 2014; Barbieri et al., 2019, 2023; Marchina et al., 2023). Thus, right-hemisphere activation may represent a structured compensatory mechanism rather than non-specific overactivation, especially when left-hemisphere language networks are severely disrupted (Nenert et al., 2017).

**FIGURE 3:**
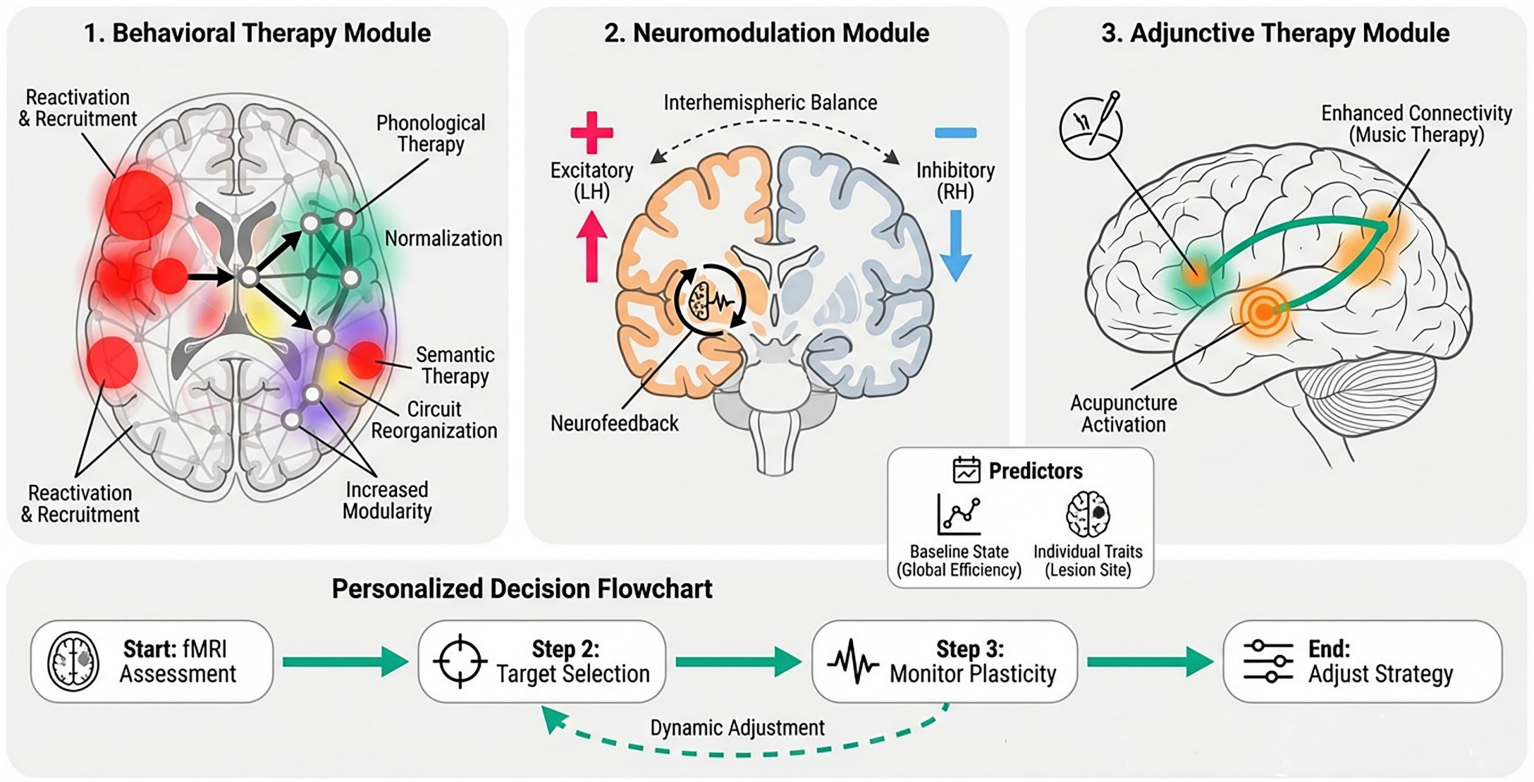
Functional magnetic resonance imaging (fMRI) findings across intervention modalities. Behavioral interventions (intensive language therapy, specific phonologic/semantic training) which promote left perilesional reactivation, domain-specific network reorganization, and enhanced global network modularity; Neuromodulation Techniques (tDCS, rTMS, and real-time fMRI neurofeedback) that target hemispheric balance, optimize interhemispheric connectivity, and train self-regulation of language networks; Adjunctive Therapies (acupuncture and music therapy) that facilitate activation of key language regions and strengthen functional connectivity.

Beyond regional and interhemispheric effects, intensive therapy further optimizes whole-brain organization. Increased resting-state modularity after imitation-based therapy is associated with improved narrative production (Duncan and Small, 2016), while language gains after comprehensive or action-based intensive therapies are linked to reorganization of attention, memory, and emotional networks, including enhanced ventral attention–memory retrieval connectivity and altered temporal dynamics in right perisylvian regions (Houldin et al., 2025; Jäger et al., 2025). Overall, intensive language therapy appears to support aphasia recovery through a staged neuroplastic process, beginning with ipsilesional reactivation and domain-general support, and progressing toward more stable inter-network coordination, right-hemisphere compensation, and whole-brain cognitive-emotional integration (Brain remodeling induced by intensive language therapy is shown in Figure 3 and Table 2).

**TABLE 2:** Functional magnetic resonance imaging (fMRI) evidence for treatment-induced brain remodeling in post-stroke aphasia.

Intervention category	Representative intervention type	Main fMRI changes reported	Neuroplastic mechanism	Clinical implication	Current limitations
Behavioral speech and language therapy	Intensive language therapy, constraint-induced language therapy, semantic/phonological training, naming therapy	Increased activation in spared left-hemisphere regions; strengthened language-network connectivity	Perilesional reactivation, strengthening of residual language circuits	Supports behavioral therapy as the core treatment approach in PSA	Effects vary by therapy intensity, chronicity, task design, and patient profile
Neuromodulation	rTMS, tDCS, fMRI neurofeedback	Modulation of local activation and interhemispheric connectivity; facilitation of left-hemisphere recruitment or suppression of maladaptive contralesional overactivity	Interhemispheric rebalancing, excitability modulation, network reconfiguration	May enhance the effects of conventional speech therapy	Optimal stimulation target, timing, dose, and patient selection remain uncertain
Combined interventions	Speech therapy plus tDCS/rTMS	More distributed connectivity changes across language and domain-general networks; possible synergistic remodeling effects	enhancement network-modulatory mechanisms	Promising for personalized rehabilitation strategies	Evidence remains limited and often based on small samples

fMRI, functional magnetic resonance imaging; PSA, post-stroke aphasia; rTMS, repetitive transcranial magnetic stimulation; tDCS, transcranial direct current stimulation.

➁ Phonological and semantic therapy

Studies of phonological component analysis have shown increased activation in left-hemisphere language regions after treatment, with brain activity shifting toward the left-dominant pattern typically observed in healthy individuals (Rochon et al., 2010). At the same time, phonological therapy also affects the right hemisphere: it can normalize overall connectivity within right-hemisphere homotopic networks while also inducing compensatory, task-specific connections that are not present in healthy controls (Truzman et al., 2021). This suggests that phonological recovery is supported by both normalization and adaptive reorganization. In terms of predictors, stronger pre-treatment resting-state activity in the right middle temporal gyrus has been associated with better phonological treatment outcomes (van Hees et al., 2014b). Post-treatment improvement has also been linked to increased activation in the left supramarginal gyrus and right precuneus (van Hees et al., 2014a), regions implicated in phonological working memory and supportive attentional processing.

Semantic therapy seems to rely more strongly on networks involved in semantic control and selection. Successful response to semantic treatment has been associated with pre-treatment activity in the left caudate nucleus (van Hees et al., 2014a), suggesting an important role for subcortical mechanisms in semantic recovery. Semantic therapy also appears to reorganize effective connectivity within the language network, particularly by restoring the central coordinating role of the left inferior frontal gyrus toward a healthier pattern (Kiran et al., 2015). Furthermore, patients with higher pre-treatment global connectivity and network efficiency within the functional semantic system—especially in frontoparietal cognitive control regions—tend to show better response to semantically oriented naming therapy (Johnson et al., 2020). These findings indicate that semantic rehabilitation depends heavily on the integrity of networks responsible for semantic monitoring, controlled retrieval, and integration.

In summary, phonological therapy relies more on auditory-motor integration, phonological working memory, and related regions such as the supramarginal gyrus, inferior parietal lobule, and right-hemisphere homologues. Whereas semantic therapy, depends more on semantic control systems, particularly the prefrontal-caudate circuit and frontoparietal control networks. These distinctions highlight the importance of aligning treatment type with the patient’s underlying neural profile (Brain remodeling induced by phonological and semantic therapy is shown in Figure 3 and Table 2).

➂ Anomia therapy

Anomia training promotes both behavioral and neuroplastic changes in chronic post-stroke aphasia. Nardo et al. (2017) revealed that both immediate phonological cuing and long-term training effects activate overlapping bilateral prefrontal networks (including the right anterior insula and inferior frontal cortex), where decreased BOLD signals reflect neural priming and enhanced processing efficiency. Johnson et al. (2021) further showed that naming therapy selectively strengthens weakened left-hemisphere temporo-frontal functional connectivity, with such restoration occurring only in behaviorally responsive patients. Together, these findings indicate that effective naming interventions improve prefrontal processing efficiency through neural priming while specifically repairing key language network impairments, closely linking neural repair with behavioral recovery and advancing our understanding of neuroplasticity in language rehabilitation (Brain remodeling induced by anomia therapy is shown in Figure 3 and Table 2).

### 6.2 Neuromodulation-based interventions

➀ Transcranial direct current stimulation (tDCS)

Current evidence indicates that tDCS induces stage-specific reorganization of language-related brain networks in post-stroke aphasia, with effects varying according to stimulation target and disease stage. In the subacute stage, cathodal cerebellar tDCS combined with speech therapy decreases whole-brain functional connectivity by weakening intra-right-hemispheric and interhemispheric interactions while reinforcing intra-left-hemispheric connectivity in core language regions (Xu X. et al., 2025). In chronic aphasia, bilateral tDCS improves functional integration within left-hemispheric language networks and enhances cross-hemispheric cooperation, particularly between the posterior cingulate cortex and right cerebellar Crus I (Marangolo et al., 2016).

Moreover, individualized fMRI-guided tDCS increases activation in bilateral and perilesional areas irrespective of stimulation polarity (Cherney et al., 2021). Anodal stimulation of the right hemisphere strengthens interhemispheric connectivity between the right inferior frontal sulcus and left frontal-temporal language regions (Alemanno et al., 2024), whereas synchronous anodal tDCS during naming tasks reduces activity in domain-general cognitive control regions, such as the bilateral anterior cingulate cortex, while promoting more efficient coordination within language networks (Darkow et al., 2017). Stage-related differences are also evident: although both acute and recovery-phase patients show enhanced bilateral frontotemporal connectivity after anodal tDCS, acute patients additionally exhibit increased temporo-occipital connectivity, whereas recovery-phase patients show reduced pre-existing compensatory hyperconnectivity in the left temporal cortex (Song et al., 2024). Collectively, these findings suggest that tDCS supports post-stroke language recovery by reshaping functional networks through modulation of interhemispheric balance, strengthening of ipsilesional integration, promotion of cross-hemispheric cooperation, and optimization of neural resource allocation (Brain remodeling induced by tDCS intervention is shown in Figure 3 and Table 2).

➁ Repetitive transcranial magnetic stimulation (rTMS)

Evidence from fMRI and fNIRS studies indicates that repetitive transcranial magnetic stimulation (rTMS) and its derivative protocols, particularly intermittent theta-burst stimulation (iTBS), facilitate recovery in post-stroke aphasia (PSA) by restoring interhemispheric balance and promoting adaptive reorganization of language-related networks. Excitatory stimulation of the left hemisphere, especially the left Broca’s area or residual language cortex, generally enhances leftward language lateralization, increases activation within ipsilesional language regions, and reduces recruitment of homologous right-hemispheric areas (Szaflarski et al., 2011; Griffis et al., 2016). In parallel, inhibitory stimulation of contralesional regions can suppress maladaptive right-hemispheric hyperactivity, with fMRI-guided target selection—such as the right inferior frontal gyrus in non-fluent aphasia and the right superior temporal gyrus in fluent aphasia—further improving therapeutic precision (Abo et al., 2012). Individualized high-frequency stimulation guided by fMRI or fNIRS has also shown favorable safety and sustained efficacy, highlighting the value of personalized neuromodulation strategies (Ohara et al., 2021; Chang et al., 2022).

At the network level, effective rTMS treatment is associated with reduced maladaptive intra-right-hemispheric connectivity, strengthened functional integration within left-hemispheric language networks, and enhanced adaptive interhemispheric cooperation (Harvey et al., 2017; Lin et al., 2022). In addition to reorganizing core language circuits, rTMS may also modulate large-scale networks such as the default mode and salience networks, with these dynamic connectivity changes relating to behavioral improvement (Szaflarski et al., 2021). iTBS, either alone or combined with behavioral therapy, appears to further optimize neural resource allocation by maintaining activation in canonical language areas while reducing recruitment of non-essential or inefficient regions (Szaflarski et al., 2018; Allendorfer et al., 2021a). It can also induce delayed changes in ventral visual and supplementary motor systems, suggesting that its therapeutic effects may extend beyond immediate language-network modulation (Allendorfer et al., 2021b). Even single-session or repeated iTBS protocols can rapidly reshape whole-brain organization, including hub centrality and semantic-network dynamics, particularly in the right hemisphere (Xu S. et al., 2021; Yang et al., 2023).

Overall, current evidence suggests that rTMS promotes language recovery in PSA through multi-level mechanisms, including suppression of maladaptive contralesional activity, facilitation of ipsilesional or residual language networks, remodeling of intra- and interhemispheric connectivity, and improvement of whole-brain network efficiency (Bai et al., 2022; Lee et al., 2022; Gan et al., 2024) (Brain remodeling induced by rTMS intervention is shown in Figure 3 and Table 2).

➂ Real-time functional MRI (fMRI) neurofeedback therapy

Real-time functional MRI neurofeedback appears to modulate language-network connectivity in post-stroke expressive aphasia, although its behavioral efficacy remains uncertain. Training targeting Broca’s area during covert language tasks has been shown to strengthen intra-left-hemispheric connectivity among frontal, temporal, central opercular, and superior parietal language modules, with the superior parietal module serving as an indirect hub that facilitates fronto-temporal integration and shifts the left language network toward a more typical organization (Sreedharan et al., 2019). Subsequent work further demonstrated that patients can learn to upregulate BOLD activity in both Broca’s and Wernicke’s areas, accompanied by increased activation in perilesional regions and right-hemispheric homologues, indicating combined local and bilateral compensatory reorganization (Sreedharan et al., 2020). However, these neuroplastic changes have not yet translated into significant improvements in naming or overall language performance. Thus, RT-fMRI neurofeedback shows promise as a tool for inducing adaptive language-network reconfiguration, but its clinical efficacy likely requires optimization of training intensity, duration, patient selection, and integration with behavioral or other neuromodulatory interventions (Brain remodeling induced by RT-fMRI neurofeedback intervention is shown in Figure 3 and Table 2).

### 6.3 Adjunctive rehabilitation strategies

➀ Acupuncture therapy

Evidence from fMRI studies demonstrates that acupuncture can promote language recovery in post-stroke aphasia by modulating activity in specific brain regions and reshaping network connectivity. Improvements in language function after acupuncture are positively correlated with increased BOLD signal activation in the left middle temporal gyrus and superior temporal gyrus (Wernicke’s area) (Chau et al., 2010). Stimulating language-related acupoints further induces activation in the left inferior frontal gyrus (opercularis, triangularis, and insula) and enhances neural responses in the left middle frontal gyrus during word-generation tasks (Li and Yang, 2011). At the network level, electroacupuncture specifically modulates both static and dynamic functional connectivity between language, salience, cerebellar, and occipital networks in patients with PSA, particularly strengthening dynamic cerebellar–language network coupling (Xu et al., 2022). Moreover, a single session of the central square array acupuncture method can enhance functionally impaired connectivity between the left middle temporal gyrus and middle frontal gyrus, while a 4-week treatment further strengthens functional connectivity between the left middle temporal gyrus and right superior temporal gyrus, changes that correlate with improved verb comprehension (Xu M. et al., 2025). Together, these findings suggest that acupuncture supports neuroplastic language recovery not only by modulating local activity in key language regions but also by promoting cross-hemispheric and multi-network functional reorganization (Brain remodeling induced by acupuncture Therapy is shown in Figure 3 and Table 2).

➁ Music therapy

Daily listening to vocal music enhanced resting-state functional connectivity between left inferior parietal lobule/central posterior gyrus and the language network compared to audiobook listening, and these increases correlated with improved verbal memory in acute stroke patients (Sihvonen et al., 2021) (Brain remodeling induced by acupuncture Therapy is shown in Figure 3 and Table 2).

➂ Mirror therapy

Mirror therapy enhances functional connectivity between left precentral gyrus, superior/middle frontal gyri, and hippocampus in acute motor aphasia, with increases correlated with improved aphasia quotient (Chen Q. et al., 2021).

### 6.4 Emerging experimental interventions: brain-computer interfaces

Brain computer interfaces (BCIs) represent an emerging yet underexplored approach for post stroke aphasia rehabilitation. Two recent studies highlight their potential. Musso et al. (2022) trained ten chronic aphasia patients using an auditory BCI with real time feedback. After 30 h of training, patients showed sustained improvements in naming, comprehension, and spontaneous speech. Resting state fMRI revealed training induced strengthening of the language network and rebalancing with the default mode network, indicating BCI driven neuroplasticity. Complementarily, Cao et al. (2025) applied motor imagery based BCI training to five patients with subacute brain injury, several of whom had aphasia. Improvements in language and cognition were observed alongside fMRI based connectomics showing enhanced connectivity across sensorimotor, language, and attention networks. Together, these findings suggest that BCI based training is a promising novel tool for aphasia recovery. The integration of fMRI allows direct assessment of neuroplastic mechanisms, providing a neurobiological foundation for future large scale trials.

### 6.5 Neuroimaging biomarkers of treatment response

A major goal of current research is to identify fMRI-based biomarkers that predict treatment response and guide individualized therapy. As summarized in Table 3, candidate biomarkers include residual left-hemisphere language activation, interhemispheric functional balance, frontoparietal network integrity, dynamic connectivity patterns, and cerebellar coupling (van Oers et al., 2010; Sims et al., 2016; Griffis et al., 2017a; Johnson et al., 2021; Pillay et al., 2018, 2022; Skipper-Kallal et al., 2017; Li and Xie, 2025; Zhang J. et al., 2022). Each biomarker measures a distinct aspect of post-stroke neuroplasticity and has been correlated with clinical language scales, though no single marker is yet robust enough for routine use.

**TABLE 3:** Functional roles of key brain regions and brain networks in post-stroke aphasia recovery.

Brain region / network	Role in language processing and recovery	fMRI findings in PSA	Potential contribution to recovery	Major controversy or unresolved issue
Left inferior frontal gyrus	Speech production, articulatory planning, syntactic processing, language control	Often hypoactive after left frontal stroke; reactivation of spared/perilesional tissue is associated with better recovery in some studies	Supports restoration of core language production mechanisms	Extent to which activation reflects true restoration versus compensatory effort remains unclear
Left posterior superior temporal gyrus	Auditory language comprehension, lexical-semantic processing	Reduced activation/connectivity is associated with comprehension deficits; partial recovery may parallel improved network integration	Supports recovery of language comprehension and auditory-verbal mapping	Recovery patterns vary by lesion extent and task type
Left middle temporal and temporoparietal regions	Lexical retrieval, semantic integration, phonological-semantic interface	Frequently involved in residual language processing and reorganization	May serve as important perilesional substrates for recovery	Their precise role may differ across aphasia subtypes
Right inferior frontal gyrus	Contralesional support for language production and control	Frequently shows increased activation in subacute and chronic PSA	May provide adaptive compensation, especially when left-hemisphere injury is extensive	Whether right frontal recruitment is adaptive or maladaptive remains a major controversy
Right temporal homologues	Semantic and auditory support under compensatory conditions	May show increased activity/connectivity in some patients	Can support residual comprehension in selected cases	Benefit appears highly dependent on lesion size, lesion site, and recovery stage
Frontoparietal executive-control network	Cognitive control, working memory, task monitoring, strategy use	Altered connectivity often accompanies language deficits and recovery	Supports therapy engagement, language control, and compensatory learning	Often underexplored relative to classical language regions
Salience network	Detection of behaviorally relevant stimuli, network switching, adaptive control	May modulate coordination between language and executive systems	Could facilitate adaptive recruitment during therapy	Direct evidence in PSA remains limited
Default mode network (DMN)	Internal mentation, semantic integration, baseline network organization	Often disrupted after stroke; may show altered coupling with language networks	May influence higher-level language and recovery efficiency indirectly	Interpretation of DMN changes in PSA remains inconsistent
Subcortical structures (thalamus, basal ganglia)	Language modulation, cortico-subcortical integration	Functional disruption can affect both language and recovery trajectories	May support language through broader circuit-level regulation	Often insufficiently characterized in fMRI studies
Cerebellum	cognitive-linguistic modulation	Increasing evidence suggests altered cerebellar connectivity in PSA	May support compensatory language processing and rehabilitation response	Still underrepresented in current fMRI literature

fMRI, functional magnetic resonance imaging; PSA, post-stroke aphasia.

Residual left-hemisphere language activation is measured as task-evoked BOLD signal change (e.g., during naming or semantic tasks) in perilesional inferior frontal, temporal, or parietal regions. It reflects restoration of the premorbid language network and perilesional plasticity. Higher activation correlates with better Western Aphasia Battery (WAB) scores and naming accuracy (DeMarco et al., 2022; Nenert et al., 2018).

Interhemispheric functional balance is often quantified using a laterality index from task-based fMRI or the ratio of left-to-right resting-state connectivity. It captures the degree of hemispheric competition or compensation. A shift toward left-lateralized activation after treatment is associated with improved language outcomes, whereas excessive right-hemisphere recruitment may correlate with poorer performance depending on lesion context (Truzman et al., 2021; Barbieri et al., 2019).

Frontoparietal network integrity is assessed via resting-state connectivity between dorsolateral prefrontal cortex and inferior parietal lobule, or graph-theoretic measures of network efficiency. This biomarker supports domain-general cognitive control (attention, working memory) that compensates for impaired language. Stronger frontoparietal connectivity predicts better treatment response and correlates with gains in naming and comprehension (Zhu et al., 2014; Zhang et al., 2021).

Cerebellar–cortical coupling is measured as resting-state connectivity between right cerebellar Crus I/II and left-hemisphere language areas (e.g., left superior temporal gyrus). It may represent error-based learning and temporal coordination of language. Stronger coupling independently predicts naming performance (Boston Naming Test) in chronic aphasia (Zhang J. et al., 2022; Li and Xie, 2025).

Collectively, these biomarkers capture complementary neuroplastic mechanisms, but none has sufficient evidence for clinical use. Future validation in large, longitudinal cohorts with standardized protocols is needed.

## 7 Controversies and competing interpretations

### 7.1 Analysis on mechanisms of right hemisphere in post-stroke aphasia rehabilitation

The role of the right hemisphere in post-stroke aphasia recovery remains a subject of ongoing debate. However, framing this issue as a simple dichotomy between adaptive compensation and maladaptive hyperactivation is overly simplistic, as right-hemisphere contributions vary considerably across regions, lesion characteristics, recovery stages, and task demands. The right inferior frontal gyrus (rIFG), the homologue of Broca’s area, is the most extensively studied region. Its increased activation may be adaptive when the left inferior frontal gyrus is extensively damaged, particularly during the subacute stage, as it supports residual speech production. Conversely, in patients with preserved left perilesional tissue, persistent rIFG hyperactivation in the chronic stage often reflects inefficient processing or maladaptive interhemispheric imbalance, especially when accompanied by poor naming performance. Right posterior temporal regions (including the superior and middle temporal gyri) serve a different function: they support compensatory semantic and auditory processing when left posterior temporal or temporoparietal areas are damaged. Their recruitment is more likely adaptive in patients with large left temporal lesions and during comprehension tasks, whereas it is not clearly maladaptive even when left temporal tissue remains intact. Right inferior parietal regions (such as the supramarginal and angular gyri) contribute to phonological working memory and semantic association. Their adaptive value is task-dependent—they may be beneficial during repetition or phonological tasks but may also reflect non-specific attention or effort rather than language-specific compensation. Lesion characteristics profoundly influence the functional significance of right-hemisphere activation. In patients with small left-hemisphere lesions and good perilesional reserve, excessive right frontal activation is often maladaptive. In contrast, when left-hemisphere language regions are severely destroyed, right-hemisphere homologues can provide necessary compensatory support. Recovery stage further modulates this relationship: right-hemisphere recruitment is frequently adaptive as a transient support mechanism in the acute and subacute stages, whereas in the chronic stage, persistent activation may be adaptive if left-hemisphere damage is irreversible, but maladaptive if it competes with residual left-hemisphere function. Therefore, the adaptive value of right-hemisphere involvement should be assessed on the basis of specific region, lesion profile, recovery stage, and functional connectivity with residual left-hemisphere networks.

### 7.2 Does recovery require left-lateralization: a perspective on complementary mechanisms

A core unresolved question is not whether left-lateralization is necessary, but rather how restitution and compensation interact as complementary mechanisms across different lesion profiles and recovery trajectories. Favorable outcomes are rarely attributable to a single pathway. In patients with small lesions and preserved perilesional tissue, recovery is primarily driven by restitution of the original left-lateralized language architecture, reflecting the reactivation of spared networks. Conversely, in patients with extensive left-hemisphere damage, compensatory pathways become more critical, with the right hemisphere and domain-general systems providing essential support. Rather than viewing restitution and compensation as competing alternatives, current evidence underscores their context-dependent complementarity: restitution predominates when feasible, whereas compensation enables communication when left-hemisphere resources are insufficient.

### 7.3 Should neuromodulation inhibit the right hemisphere or enhance the left

The optimal stimulation target in PSA remains controversial. The traditional approach of inhibiting right frontal homologues is grounded in the interhemispheric competition model, whereas alternative approaches seek to enhance residual left perilesional networks or tailor stimulation according to lesion topology. The heterogeneity of imaging findings suggests that personalized stimulation strategies will likely be more effective than one-size-fits-all protocols.

## 8 Current research gaps

Despite substantial progress, several major research gaps continue to limit the field.

First, sample sizes remain small in many fMRI studies, reducing statistical power and external validity. Second, patient populations are highly heterogeneous with respect to aphasia subtype, lesion location, lesion volume, vascular status, cognitive reserve, and rehabilitation exposure. Third, many studies remain cross-sectional, whereas longitudinal within-subject designs are essential for capturing the dynamic evolution of recovery. Fourth, relatively few investigations integrate functional imaging with structural connectivity, perfusion imaging, lesion network mapping, or computational modeling. Fifth, the relationship between neural change and clinically meaningful communication outcomes remains insufficiently resolved. Sixth, Finally, there is still no widely accepted imaging framework for predicting which patients will benefit from which intervention at which stage of recovery.

## 9 Discussion

An important conclusion of this review is that methodological heterogeneity remains a major barrier to translating fMRI findings into clinically useful biomarkers for post-stroke aphasia. Differences in task paradigms, acquisition parameters, preprocessing pipelines, lesion normalization, connectivity metrics, parcellation schemes, and motion-control procedures may partly explain inconsistent results across studies. This issue is particularly relevant in aphasia because language performance during scanning, lesion anatomy, altered neurovascular coupling, and patient motion can all influence BOLD activation and connectivity estimates.

Despite these methodological challenges, the review also arrives at several consistent and clinically informative insights regarding the nature of post-stroke aphasia (PSA) recovery. PSA recovery is best understood as a dynamic, multi-stage, and network-based neuroplastic process rather than as the simple re-emergence of function within isolated language regions. fMRI studies consistently show that stroke-induced aphasia involves disruption not only of classical language areas but also of distributed language-related and domain-general systems. Across the recovery trajectory, activation patterns and functional connectivity evolve from early inhibition and instability to broader compensatory recruitment and, in some patients, more efficient long-term reorganization.

A central insight emerging from the current literature is that no single neural pathway of recovery applies to all patients. Instead, the balance among left perilesional restoration, contralesional compensation, domain-general support, and cerebellar or subcortical participation appears to vary according to lesion severity, residual structural integrity, time post-stroke, and treatment exposure. This helps explain why apparently conflicting imaging findings often coexist in the literature: different studies may be capturing different recovery routes in different patient populations. At the same time, important controversies remain unresolved. The functional significance of right-hemisphere activation is likely conditional rather than absolute. Broad bilateral activation may signal adaptive compensation during one phase or under one lesion profile, but may reflect inefficiency in another.

From a translational perspective, the most promising direction for the field lies in the development of biomarker-guided rehabilitation strategies. Therefore, future research should move toward standardized reporting and harmonized imaging protocols. Multimodal imaging approaches that combine fMRI with structural connectivity, perfusion imaging, lesion network mapping, and detailed behavioral assessment may improve the biological validity of fMRI markers. Large-scale longitudinal studies using transparent preprocessing pipelines and clinically meaningful outcome measures are needed to determine which imaging features reliably predict spontaneous recovery and treatment response. Achieving this goal will require larger multicenter longitudinal studies, standardized imaging and behavioral protocols, multimodal data integration, and closer alignment between neuroimaging outcomes and clinically meaningful language function, with the ultimate aim of stratifying patients by likely recovery pathway and guiding stage-specific intervention choices.

It is important to consider the clinical characteristics of patients in the cited studies. As summarized across the literature, patient age ranged from approximately 30 to 80 years, stroke types included both ischemic (majority) and hemorrhagic cases, lesion locations varied widely (frontal, temporoparietal, subcortical, and mixed), and recovery stages spanned acute (<2 weeks), subacute (2 weeks–6 months), and chronic (>6 months) phases. Sample sizes varied considerably, from small pilot studies (6–15 cases) to larger cohorts (40–70 cases). These variables—particularly lesion site, stroke type, and time since stroke—can substantially influence fMRI findings and generalizability, while small sample sizes limit statistical weight and replicability. A more nuanced consideration of lesion location and stroke type is warranted. Cortical strokes involving left perisylvian regions typically cause severe deficits, with recovery relying on perilesional reactivation and right hemisphere compensation. Subcortical strokes (e.g., basal ganglia, thalamus) disrupt language indirectly via cortico subcortical circuits; fMRI shows preserved cortical activation but altered connectivity, and recovery depends more on strengthening left hemisphere fronto temporal connectivity than on extensive right hemisphere recruitment (Xu et al., 2020; Zhang J. et al., 2022). Stroke type also matters: ischemic strokes cause focal injury with penumbra, allowing dynamic BOLD changes over time (Wang et al., 2025b); hemorrhagic strokes involve mass effect and diaschisis, affecting reorganization timing and magnitude, though limited data preclude firm conclusions (Boren et al., 2023). Future studies should stratify patients by lesion topography (cortical, subcortical, or mixed) and etiology (ischemic vs. hemorrhagic) to identify distinct recovery trajectories and guide targeted rehabilitation.

## 10 Conclusion

Based on current fMRI evidence, recovery from post-stroke aphasia reflects a dynamic process of large-scale brain reorganization involving stage-specific shifts in regional activation, network connectivity, and cross-network coordination. The acute phase is dominated by widespread functional disruption and early compensatory responses; the subacute phase often shows peak bilateral recruitment and high neuroplastic potential; and the chronic phase is characterized by more stable, individualized patterns of reorganization. In many patients, successful recovery is closely associated with restoration or efficient reorganization of residual left-hemisphere language networks, while the right hemisphere and cerebellum may provide important support under specific structural and functional constraints.

Looking forward, the integration of multimodal neuroimaging and precision rehabilitation frameworks may substantially improve our understanding of PSA recovery and facilitate the development of individualized, stage-adapted treatment strategies.
